# Cellular and molecular phenotypes depending upon the RNA repair system RtcAB of *Escherichia coli*

**DOI:** 10.1093/nar/gkw628

**Published:** 2016-07-08

**Authors:** Christoph Engl, Jorrit Schaefer, Ioly Kotta-Loizou, Martin Buck

**Affiliations:** 1 Faculty of Natural Sciences, Division of Cell & Molecular Biology, Imperial College London, London SW7 2AZ, UK; 2 Institute for Global Food Security, Queen's University Belfast, Belfast BT9 7BL, UK

## Abstract

RNA ligases function pervasively across the three kingdoms of life for RNA repair, splicing and can be stress induced. The RtcB protein (also HSPC117, C22orf28, FAAP and D10Wsu52e) is one such conserved ligase, involved in tRNA and mRNA splicing. However, its physiological role is poorly described, especially in bacteria. We now show in *Escherichia coli* bacteria that the RtcR activated *rtcAB* genes function for ribosome homeostasis involving rRNA stability. Expression of *rtcAB* is activated by agents and genetic lesions which impair the translation apparatus or may cause oxidative damage in the cell. Rtc helps the cell to survive challenges to the translation apparatus, including ribosome targeting antibiotics. Further, loss of Rtc causes profound changes in chemotaxis and motility. Together, our data suggest that the Rtc system is part of a previously unrecognized adaptive response linking ribosome homeostasis with basic cell physiology and behaviour.

## INTRODUCTION

Many bacteria contain a small operon *rtcBA* encoding the RtcAB proteins whose biochemical characterizations from a range of sources show they enzymatically modify RNA ends (RtcA) and carry out ligation (RtcB) of these ends respectively (1–4). In eukaryotes and archaea a role for RtcB in tRNA splicing and HAC1/XBP1 mRNA splicing for the unfolded protein response has emerged (5–10). The absence of introns in *Escherichia coli* tRNAs suggest a wider role for the bacterial Rtc system than currently documented from eukaryotic and archaeal studies (11,12).

Loss of RtcB creates large morphological changes along development pathways outside of the unfolded protein response and tRNA maturation (9). RtcA- and RtcB-dependent morphological changes include inhibition of post traumatic axon regeneration in the central nervous system in *Drosophila* and *Caenorhabditis elegans*, respectively (13,14). To determine the role of the RtcR activated *rtcBA* operon in *E. coli* bacteria we examined phenotypes and transcriptional profiles of cells lacking RtcA and RtcB, and determined conditions whereby RtcR was activated to drive elevated expression of *rtcBA*. Our findings suggest that in *E. coli* the Rtc system supports key cellular processes ranging from maintaining the translational apparatus to control of antibiotic sensitivity and chemotactical behaviour.

## MATERIALS AND METHODS

### Bacterial strains and genetic manipulations

Unless stated otherwise, bacteria were grown in LB or M9 medium as specified at 37°C with appropriate antibiotics. In frame deletions of *rtcR, rtcB* and *rtcA* in *E. coli* BW25113 were from the Keio collection (15) and transduced into *E. coli* MG1655 for study. The mRNA expression levels of *rtcB* and *rtcA* in the cells lacking *rtcB* and *rtcA* were assessed by real-time RT-qPCR (Supplementary Figure S1a). The VapC_LT2_ gene was synthesized by Thermo Fisher Scientific GENEART GmbH (Germany) and subcloned into pBAD18cm. The genes encoding *rtcR_ΔNTD_, rtcB* and *rtcA* were amplified from the *E. coli* MG1655 chromosome and subcloned into pBAD18cm. The *rtcA*_H308A_ and *rtcB*_H337A_ catalytic mutants were constructed using the QuikChange Site-Directed Mutagenesis kit (Agilent) according to the instructions of the manufacturer. The *rtcBA* promoter including regulatory sequences (175 nt upstream of the *rtcB* start codon) was synthesized by Thermo Fisher Scientific GENEART GmbH (Germany) and subcloned into pBBR1MCS-4 containing *gfp-mut3* or *lacZ* including a *rbs30* ribosome binding site (*rbs30*: TCTAGAGATTAAAGAGGAGAAATACTAGATG; from Registry of Standard Biological Parts, http://partsregistry.org) and a transcriptional terminator (16,17). Antibiotic concentrations: Ampicillin: 100 μg/ml; Chloramphenicol: 25 μg/ml.

### β-Galactosidase assay

Cells containing the pBBR1MCS-4(P*_rtcBA_-lacZ*) reporter were grown at 37°C in LB broth containing the appropriate antibiotic. Expression of VapC_LT2_ and RtcR_ΔNTD_ from pBAD18cm was induced for 1h with 0.02% L-arabinose. LacZ activity was measured at mid-log phase as described (18).

### Motility assay

Motility assays were performed as described (19). About 2 μl of bacterial culture were spotted onto soft agar plates supplemented with the appropriate antibiotics and 0.2% L-arabinose. Motility was measured as the diameter of bacterial spread in mm after overnight incubation at room temperature.

### Survival assays

For survival assays, optical density at 600 nm (OD_600_) of the bacterial cultures was recorded in absence and presence of stress. Stress conditions (VapC_LT2_, colicin D, tetracycline) were introduced at mid-log phase of growth. Expression of VapC_LT2_ from pBAD18cm was induced by 0.02% L-arabinose, colicin D and tetracycline were added to the bacterial cultures at concentrations of 250 nM and 1.5 μg/ml, respectively.

### Screens for *rtcBA* inducing genetic lesions and abiotic compounds

Genetic lesions inducing *rtcBA* expressions were screened by transforming a pool of Keio mutants (15) and a small peptide/ small RNA mutant library (20) with pBBR1MCS-4(P*_rtcBA_-lacZ*) and subsequent blue/white screening on XGal plates. Positive clones were then subjected to β-Galactosidase assays in liquid culture. To screen for abiotic *rtcBA* inducers cells containing pBBR1MCS-4(P*_rtcBA_-gfp*) grown in M9 medium were resuspended in Phenotype MicroArray plates (Biolog Inc., USA) and transferred to black 96-well clear-bottom tissue culture plates. In a BMG FLUOstar Omega microplate reader (BMG Labtech Ltd., UK) OD_600_ and green fluorescence (excitation: 485 nm; emission: 520 ± 10 nm, gain: 1000) were measured and promoter activity was expressed as fluorescence emission EM_520_ per OD_600_.

### Inverse PCR

Inverse polymerase chain reaction (PCR) as described in (21) was used to identify selected genetic lesions which increased *rtcBA* expression.

### RNA deep sequencing

RNA deep sequencing of whole cells was as described (21). For RNA deep sequencing of ribosome fractions first-strand cDNA synthesis was primed with a N6 randomized primer. After fragmentation, the Illumina TruSeq sequencing adapters were ligated in a strand specific manner to the 5′ and 3′ ends of the cDNA fragments. This way, a strand specific PCR amplification of the cDNA was achieved using a proof reading enzyme. The cDNA was purified using the Agencourt AMPure XP kit (Beckman Coulter Genomics). The cDNA samples were pooled for near equimolar amounts and single-end sequenced (75 bp) on an Illumina NextSeq 500 system. The cDNA reads were analysed via the RNA-seq workflow within Partek^®^ Genomics suite 6.6, including a QA/QC step to gauge the sequencing quality. Each sample yielded close to equivalent total reads aligned to the *E. coli* K-12 reference genome CP009273. The experiments were performed in duplicate. Gene ontology (GO) enrichment analysis was performed using the PANTHER Classification System (22).

### Ribosome profiling

Profiling was conducted under ribosome-associative conditions. Cells were grown with shaking in 500 ml M9 in a 2 l flask supplemented with 0.02% L-arabinose and the appropriate antibiotic and harvested at mid-log phase (OD_600_ ∼ 0.5). Cell pellets were resuspended in sterile ribosome buffer (20 mM HEPES-KOH, pH 7.5, 6 mM magnesium acetate, 30 mM ammonium chloride, 4 mM 2-mercaptoethanol, 0.1 unit/μl DNAse) containing 0.5 mg/ml lysozyme and complete protease inhibitor (1–2 tablets/10 ml) and frozen overnight at −80C. The volume was adjusted to normalize for OD_600_. After sonication, cell debris was spun down (5000 rpm; 15 min; 4°C), supernatant loaded onto a 4 ml 37.6% sucrose cushion (in ribosome buffer) and ultracentrifuged (31 000 rpm; 2.5 h; 4°C). The pellet containing the ribosomes was resuspended in 200 μl ribosome buffer and the ribosomes clarified further (5000 rpm; 15 min; 4°C). The supernatant was layered onto a 10–40% sucrose gradient (in ribosome buffer) and ultracentrifuged (35 000 rpm; 3 h; 4°C). Ribosomal fractions were collected after piercing the bottom of the tube and dripping into wells of a microtiter plate. Adsorption of the fractions at 260 nm was recorded using a spectrophotometer. RNA from the fractions was isolated via peqGOLD TriFast FL reagent (PEQLAB) and inspected by capillary electrophoresis on a Shimadzu MultiNA microchip electrophoresis system. The isolated RNA was subjected to Illumina TruSeq sequencing as described in RNA sequencing.

### Real-Time quantitative PCR

Total bacterial RNA was extracted using the Qiagen RNeasy Protect Bacteria mini kit and treated with DNase I (Promega) and reverse transcription was performed using SuperScript III Reverse Transcriptase. The RT-qPCR assays were performed in the OneStepPlus Real-Time qPCR System (Applied Biosystems) using the Power SYBR Green PCR Master Mix (Applied Biosystems). The *rtcB* mRNA, *rtcA* mRNA, 23S rRNA, 16S rRNA and 5S rRNA sequences were amplified using the target-specific primer pairs 5′-ACG TGA TAA AGG TGC CTG GG-3′ and 5′-CAC ACC TGG TCC GAC TCA TC-3′; 5′-GAC CAA CTG GTG CTA CCG AT-3′ and 5′-GCG TTA CGC CAT CTG TTT CT-3′; 5′-AGA GTA ACG GAG GAG CAC GA-3′ and 5′-CAC TAT GAC CTG CTT TCG CA-3′; 5′-CGG ACG GGT GAG TAA TGT CT-3′ and 5′-CTC AGA CCA GCT AGG GAT CG-3′; 5′-GGT GGT CCC ACC TGA CCC-3′ and 5′-ATG CCT GGC AGT TCC CTA CT-3′, respectively. The 5S rRNA served as an endogenous control.

## RESULTS AND DISCUSSION

### The *Escherichia coli* Rtc system responds to challenges to the translation apparatus

**Figure 1.**
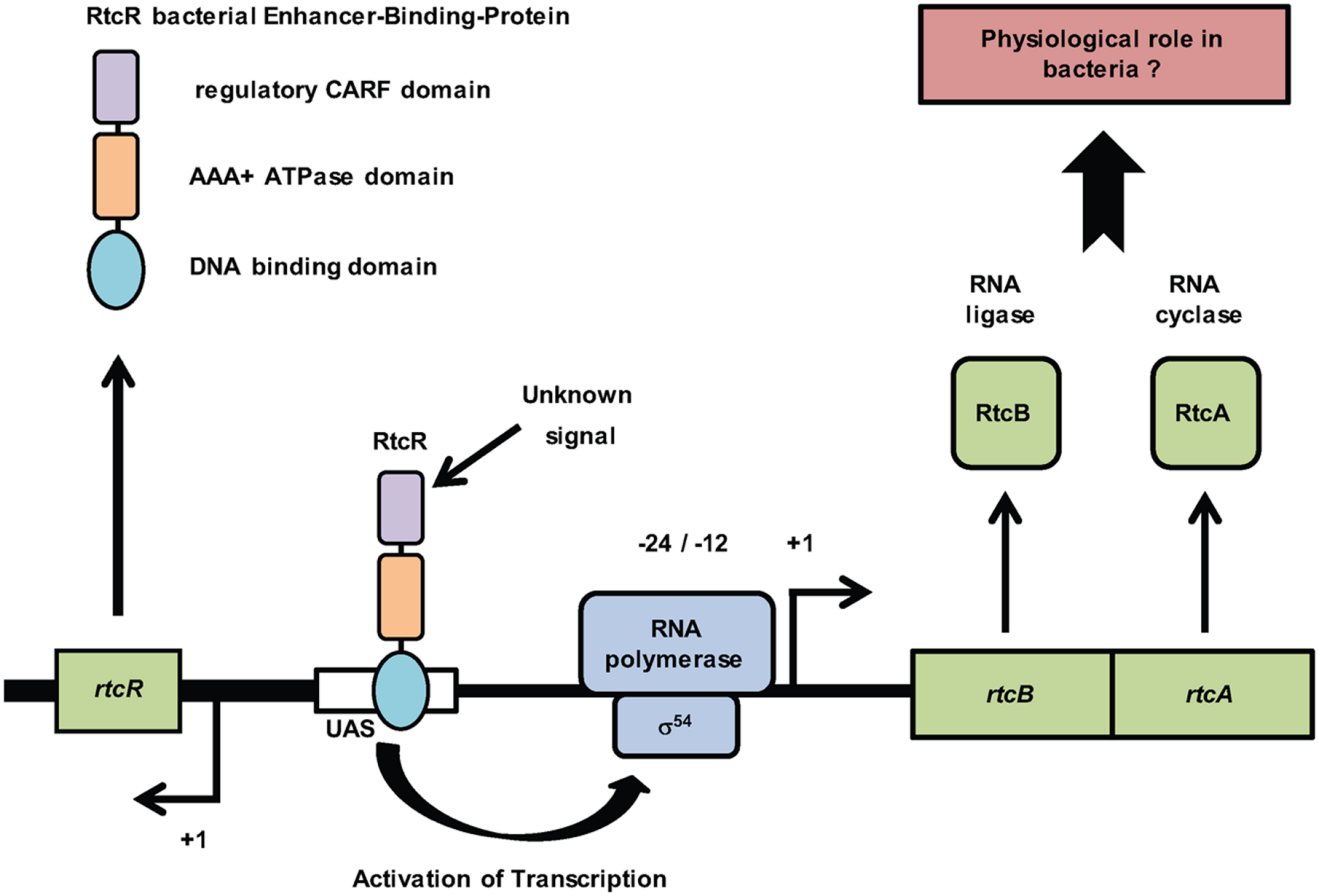
The *rtc* locus in *Escherichia coli*. Expression of the *rtcBA* operon is positively activated at the transcription level by the bacterial enhancer-binding-protein RtcR working through the σ^54^-RNA polymerase. RtcR binds to the upstream-activating-sequence (UAS) upstream of the *rtcBA* promoter (−24/−12 sequences) and transduces an unknown signal via its CARF domain to σ^54^-RNA polymerase causing upregulation of *rtcBA* transcription. RtcBA encode a RNA repair system (RtcA: RNA cyclase; RtcB: RNA ligase) whose physiological role is explored here.

Expression of the *rtcBA* operon is activated at the transcription level by the enhancer binding protein RtcR working through the σ^54^ RNA polymerase (1). RtcR is a CARF domain containing protein (23) and transduces an unknown signal to cause upregulation of *rtcBA* transcription (Figure 1 and Supplementary Figure S1b). RtcR_ΔNTD_, a N-terminally truncated form of RtcR lacking the regulatory CARF domain, was previously shown to be constitutively active in inducing *rtcBA* expression (1). In light of Rtc's role in eukaryotic and archaeal tRNA maturation we tested whether tRNA breaks could induce *rtcBA* in *E. coli*. The ribotoxin VapC_LT2_ is a tRNase from *Salmonella enterica* serovar Typhimurium LT2 targeting initiator tRNA^fMet^ (24). VapC_LT2_ thereby inhibits translation and as a consequence causes cell growth to cease (24).

**Figure 2.**
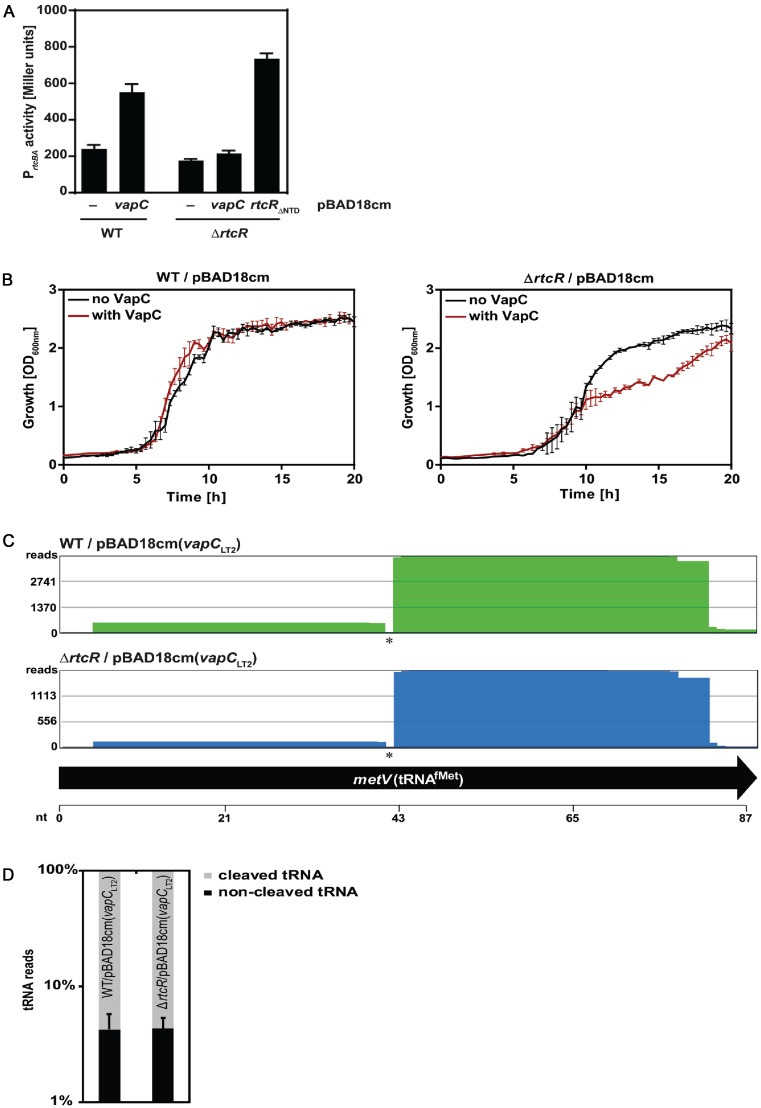
The *Escherichia coli* Rtc system responds to tRNA damage. (**A**) Activity of the *rtcBA* promoter measured in Miller units in wild-type (WT) and in cells lacking the activator RtcR (Δ*rtcR*). Cells contain empty pBAD18cm (−), pBAD18cm expressing VapC_LT2_ (*vapC*) or a constitutively active RtcR variant (*rtcR*_ΔNTD_). (**B**) Growth of WT and Δ*rtcR* cells in absence (black) and presence (brown) of VapC_LT2_. Expression of VapC_LT2_ from pBAD18cm was induced at exponential phase. (**C**) RNA deep sequencing of tRNA^fMet^ (here: *metV*) of WT and Δ*rtcR* cells producing VapC_LT2_. The distribution of all reads for tRNA^fMet^ is presented and cleavage of tRNA^fMet^ at the anticodon loop is indicated by asterisks. (**D**) Percentage of cleaved and non-cleaved reads at the tRNA^fMet^ anticodon loop of WT and Δ*rtcR* cells producing VapC_LT2_.

Indeed, ectopic production of VapC_LT2_ upregulated the activity of the *rtcBA* promoter in an RtcR-dependent manner (Figure 2A). Moreover, the growth inhibiting effect of VapC_LT2_ was more pronounced in cells lacking *rtcR* compared to wildtype (WT) cells (Figure 2B) demonstrating that the Rtc system counteracts the toxic effect of VapC_LT2._ We examined whether Rtc acts as an RNA ligase to directly re-ligate the cleaved tRNA^fMet^. The cleavage site of VapC_LT2_ has been mapped to nucleotides +38/+39 in the anticodon stem loop of tRNA^fMet^ (24). Using RNA deep sequencing we were able to detect this tRNA^fMet^ cleavage event in presence of VapC_LT2_ (Figure 2C asterisk). Rtc-dependent healing of the tRNA^fMet^ breaks however was not apparent (Figure 2C and D), probably because the expression of RtcB is not completely abolished in the cells lacking *rtcR* (Supplementary Figure S1b). Any modest changes in re-ligation between the cells lacking *rtcR* and the WT cells, which would result in the observed differences in growth, were not detected by the methodology employed. The presence of the cleaved tRNA^fMet^ when VapC leads to increased *rtcBA* promoter activity is consistent with the cleaved tRNA^fMet^ acting as stressor for activation of the *rtcBA* promoter.

To test whether the Rtc response was specific to broken initiator tRNA^fMet^ we measured *rtc*-dependent survival in presence of colicin D, a ribotoxin targeting the anticodon loop of elongator tRNA^Arg^ (25). Again, cells lacking *rtcA* and *rtcB* were less able to withstand the stress imposed by colicin D than WT or complemented cells (Supplementary Figure S2). We conclude the Rtc system appears to (i) mount responses to tRNases and (ii) not simply repair damaged tRNAs suggesting other roles for Rtc in these cells potentially linked to effects that broken tRNAs may have on the functioning of the translation apparatus.

We next performed unbiased screens for abiotic stressors and mutants which caused up-regulation of the *rtcBA* promoter. We found that the Rtc system is activated by agents (Supplementary Figure S3a and Supplementary Table S1) or genetic lesions (Supplementary Figure S3c and Supplementary Table S1) which impair the translation apparatus or may cause oxidative damage in the cell. Selected abiotic compounds and genetic lesions were shown not to increase the chromosomal *gfp* and *lacZ^+^* expression respectively (Supplementary Figure S3b and c), confirming the specificity of the activation of the *rtcBA* operon. Notably, several studies suggest that ribosomal RNA is a major target for oxidative damage (26,27). Further, the stress signalling to RtcR is rather specific since numerous other challenges to cells did not cause up-regulation of the *rtcBA* genes (Supplementary Figure S3a). As with VapC_LT2_ and colicin D, survival of a tetracycline challenge, a ribosome-targeting antibiotic which induced Rtc, was impaired in absence of a functional Rtc system (Supplementary Figure S4).

Taken together, our data suggest that the Rtc system is a helpful adaptive response to challenges to the translation apparatus. A distinct single molecular target for Rtc induction however is not so evident; instead, Rtc inducing challenges act on multiple levels within the translation apparatus: (i) tRNA stability and editing, (ii) interaction of amino-acyl-tRNA with the 30S ribosomal subunit and (iii) peptidyl transferase activity of the 50S ribosomal subunit. Significantly, our findings suggest a novel response of bacteria to antibiotics exposure.

### The *Escherichia coli* Rtc system functions in ribosome homeostasis and chemotaxis

**Figure 3.**
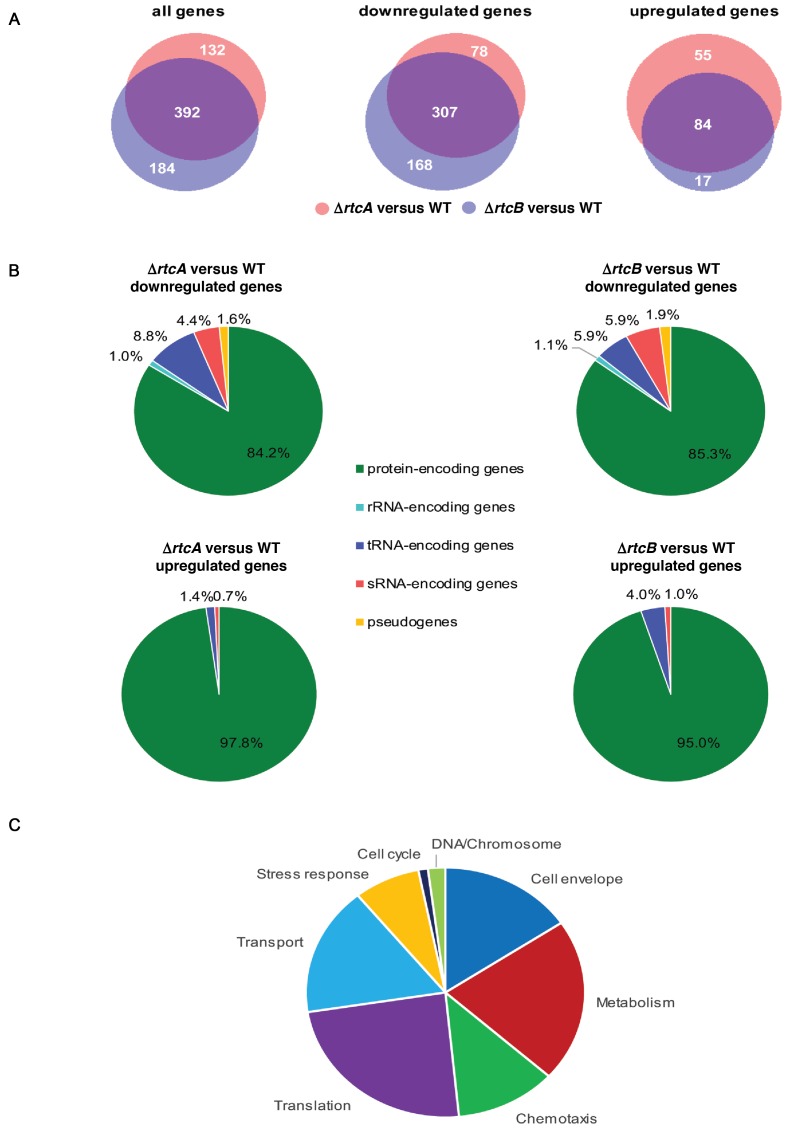
The *Escherichia coli* Rtc system is operational without exogenous stress. RNA deep sequencing of conventionally grown *E. coli* WT and cells lacking RtcA (Δ*rtcA*) or RtcB (Δ*rtcB*). Depicted are (**A**) area-proportioned Venn diagrams of genes differentially expressed in Δ*rtcA* and Δ*rtcB* compared to WT at least 4-fold (*P*-value < 0.01) and (**B**) pie charts illustrating the general functional roles of these genes. (**C**) Depicted is also the functional distribution of genes at least 4-fold differentially expressed in Δ*rtcA* and/or Δ*rtcB* compared to WT.

We sought evidence for Rtc activity in the absence of genetic lesions or any applied abiotic stress. Indeed, RNA deep sequencing of cells growing in conventional growth media but lacking *rtcA* or *rtcB* revealed Rtc-dependent changes in the transcriptome demonstrating that the Rtc system was operating in conventionally cultured WT cells without any exogenous stress (Figure 3 and supplementary MS Excel spreadsheet). A total of 708 genes were at least 4-fold differently expressed in an Rtc-dependent manner (cut-off: log_2_(*Δrtc* [RPKM]/WT [RPKM]) > +2 or < −2; *P*-value < 0.01). In total, 524 and 576 genes are differentially expressed in cells lacking *rtcA* and *rtcB* compared to the WT and 392 of these genes are common for both mutants (Figure 3A). The majority of genes are downregulated in cells lacking *rtcA* and *rtcB* in comparison to the WT. Approximately 15% of the downregulated genes are non-protein encoding genes, i.e. rRNA, tRNA and sRNA encoding genes together with pseudogenes, while the respective percentage for the upregulated genes is <5% (Figure 3B). This observation is consistent with the role of the Rtc system in RNA repair. Approximately 30% of the differentially regulated genes (the largest functional sub-group) map directly to the ribosome (e.g. genes encoding rRNAs, tRNAs and ribosomal proteins) or function in amino acid biosynthesis and transport, strengthening the link between Rtc activity and the translation apparatus (Figure 3C). Further, many of the differentially expressed genes have a role in redox, iron-sulphur and nucleotide metabolism as well as in responses to oxidative stress and DNA damage (Supplementary MS Excel spreadsheet). Among these is *yobF* whose deletion increased expression of *rtcBA*. GO enrichment analysis performed using the PANTHER classification system confirmed that genes associated with the chemotaxis and motility, metabolic and catalytic processes, ion and nucleotide binding together with the ribosome appear to be significantly enriched or depleted (Table 1). The RNA deep sequencing signatures also indicate an unexpected role for Rtc in chemotaxis and motility affecting the expression of chemotaxis receptors and regulators as well as flagellar components.

**Table 1.** The *Escherichia coli* Rtc system is linked to the translation apparatus and cell motility

	Percentage of genes	Fold enrichment	*P*-value
GO biological process			
Chemotaxis	2.8%	4.29	8.89 × 10^−04^
Cell motility	5.1%	4.13	7.61 × 10^−08^
Metabolic process	47.6%	0.78	3.19 × 10^−08^
GO molecular function			
Structural constituent of ribosome	4.1%	3.10	9.14 × 10^−04^
Catalytic activity	30.8%	0.65	4.80 × 10^−14^
Ion binding	17.4%	0.56	7.67 × 10^−12^
Nucleotide binding	8.3%	0.51	5.57 × 10^−06^
GO cellular component			
Bacterial-type flagellum	3.6%	5.18	1.09 × 10^−07^
Ribosome	4.3%	3.01	1.51 × 10^−04^
Membrane^a^	41.9%	1.26	1.39 × 10^−02^

^a^The association with the membrane is statistically significant only in Δ*rtcA* versus WT.

Gene ontology (GO) enrichment analysis of the 603 genes with known function differentially regulated in ΔrtcA versus WT, ΔrtcB versus WT or both. WT: wild-type *Escherichia coli*.

**Figure 4.**
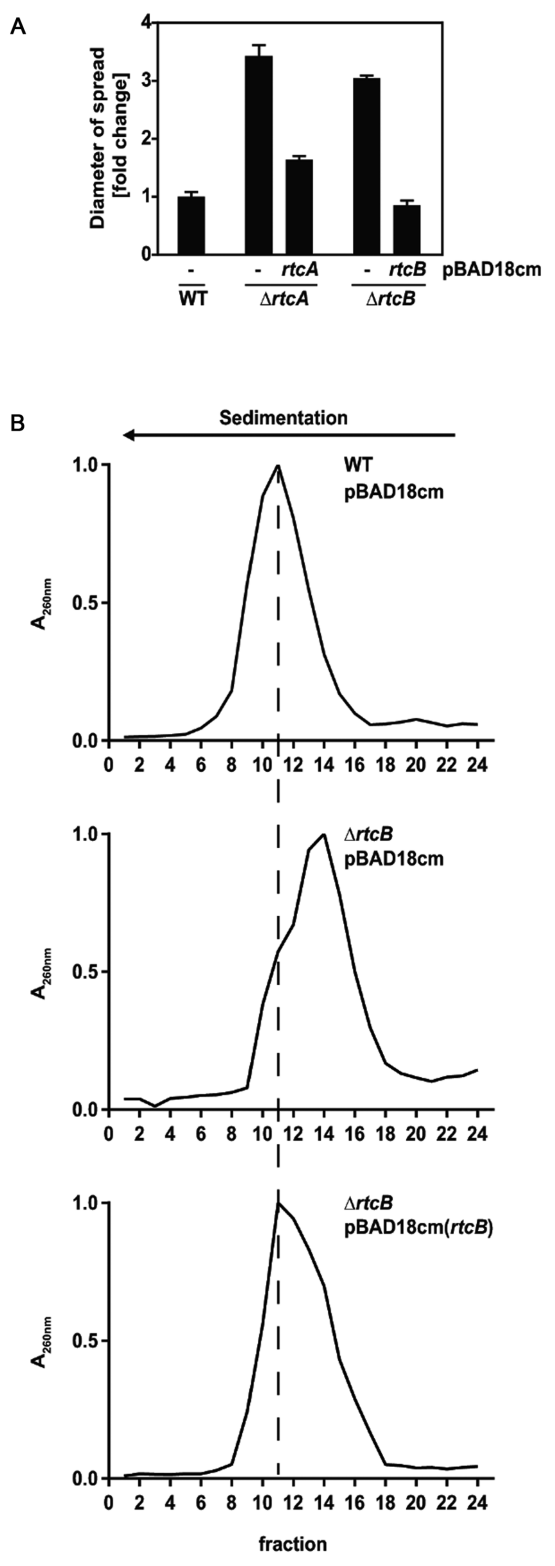
The *Escherichia coli* Rtc system affects motility and ribosome homeostasis. (**A**) Motility was assessed on soft agar plates and measured as the diameter of bacterial spread. Shown are fold changes with respect to WT. Cells contained empty pBAD18cm (−), pBAD18cm expressing RtcA (*rtcA*) or RtcB (*rtcB*). (**B**) Ribosome profiles were extracted under ribosome-associative conditions at exponential phase from conventionally grown WT/pBAD18cm, Δ*rtcB*/pBAD18cm together with complemented Δ*rtcB*/pBAD18cm(*rtcB*) cells. Ribosomal RNAs in fractions were measured at A_260_.

**Figure 5.**
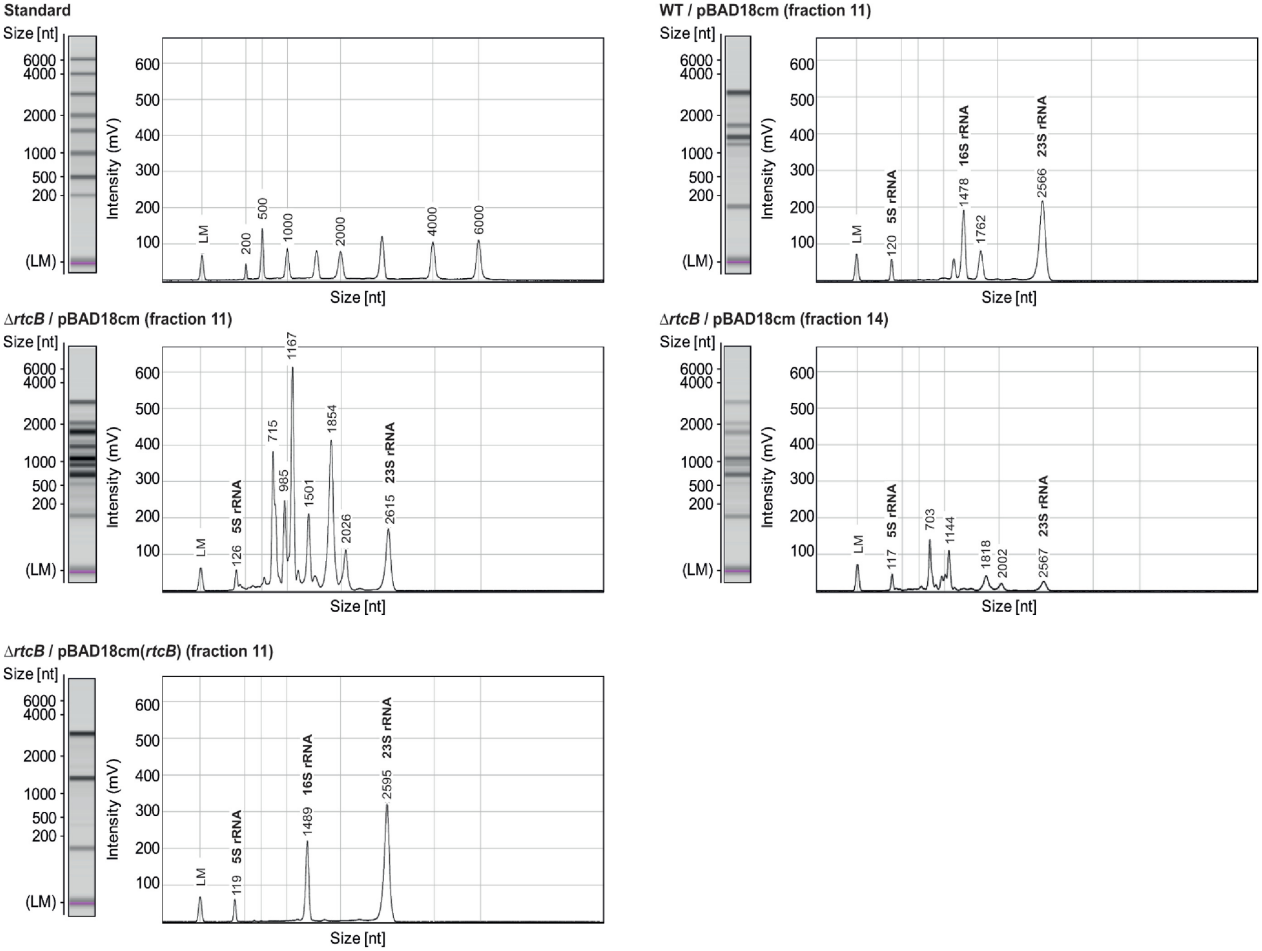
The *Escherichia coli* Rtc system stabilizes rRNA. Ribosomal RNAs from selected fractions taken during ribosome profiling were analysed via capillary electrophoresis.

**Figure 6.**
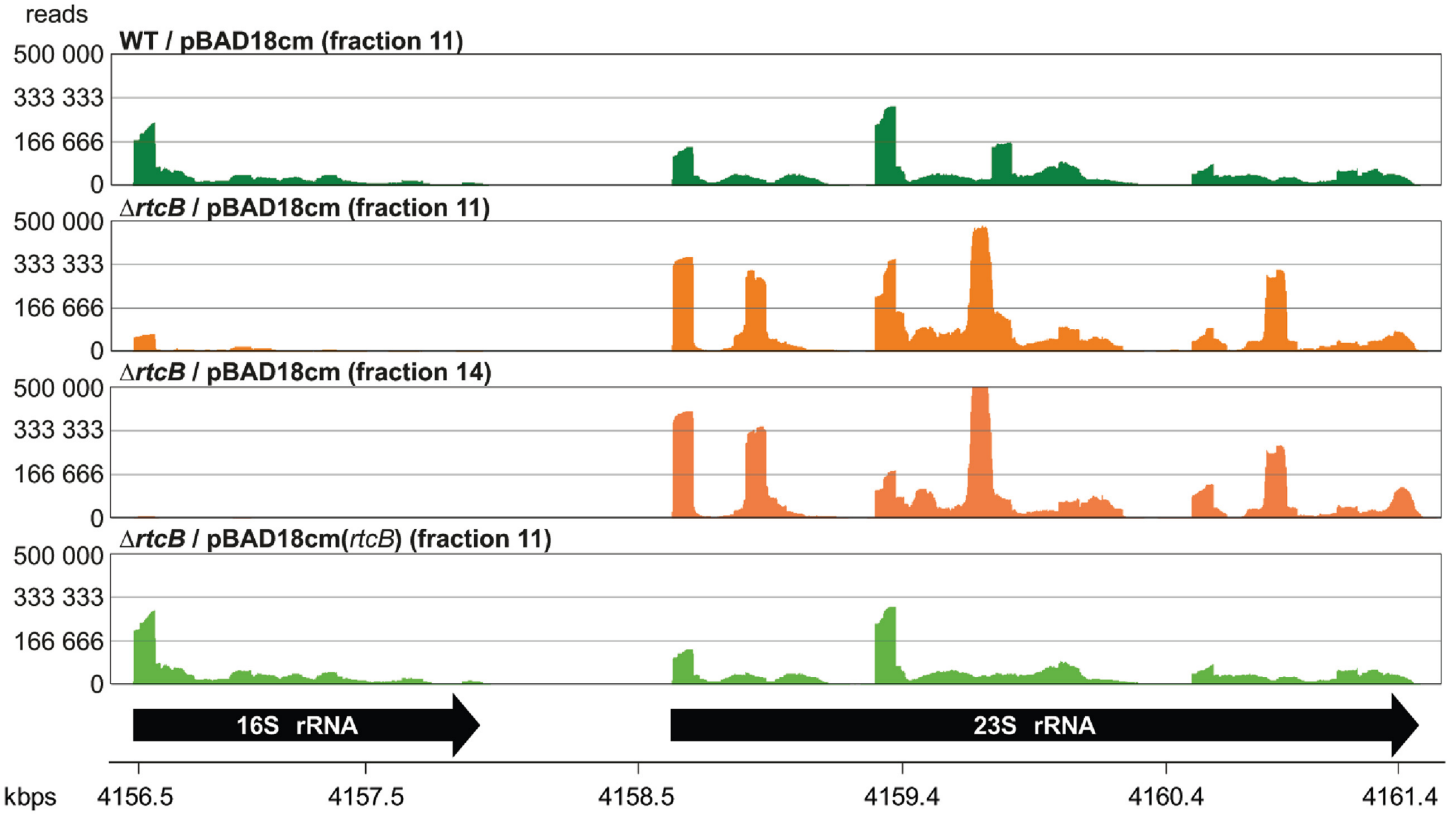
The *Escherichia coli* Rtc system affects 16S rRNA levels. Ribosomal RNAs from selected fractions taken during ribosome profiling were subjected to Illumina Truseq sequencing. Shown is the RNA deep sequencing signature of the *rrnB* operon, representative for all other *rrn* operons identified.

To directly test the apparent impact of the Rtc system on chemotaxis and the translation apparatus we performed motility assays on soft agar plates as well as ribosome profiling. Indeed, both motility and ribosome profiles of *rtc* mutants were distinct from those of WT cells (Figure 4). In line with the increased expression of chemotaxis and motility genes, in soft agar tests the diameter of spread of cells lacking *rtcA* or *rtcB* was 3-fold increased (Figure 4A), while ribosomes from cells lacking *rtcB* sedimented slower than those of WT cells (Figure 4B). Importantly, complementation with ectopically expressed RtcA and/or RtcB rescued the mutant phenotypes confirming that the observations can be attributed to the action of the Rtc system. We reasoned that the changes in the ribosome profile might be associated with the reported role of Rtc in RNA metabolism (1–4) and therefore examined several fractions across the ribosome profiles for their RNA content. Ribosome fractions from cells lacking *rtcB* indeed showed marked degradation of rRNAs while complementing Δ*rtcB* with ectopically expressed RtcB stabilized the rRNAs above the level of WT cells (Figure 5). The ectopically expressed *rtcB* mRNA levels were shown by RT-qPCR to be more than 10-fold higher than those in WT cells and this over-expression is most likely responsible for the observed stabilization of the 16S rRNA (Supplementary Figure S1b). Moreover, RNA deep sequencing revealed that the ribosome fractions of Δ*rtcB* cells contained significantly less 16S rRNA than WT or complemented cells, marked differences in 23S rRNA levels however were not evident (Figure 6). RT-qPCR assessment of 16S and 23S rRNA levels in WT, Δ*rtcB* and complemented cells confirmed these results (Supplementary Figure S5).

## CONCLUSION

In summary, the evidence presented here around the cellular and molecular phenotypes associated with the loss of Rtc supports a widening physiological role for RNA repair systems in bacteria far beyond for RtcB's classical role in ligating tRNA parts, an activity which may be used in *E. coli* in response to ribotoxins but not directly for tRNA biogenesis. Importantly, some antibiotics targeting the translational apparatus are more effective when Rtc is not functional, demonstrating that the Rtc system can be a part of the native resistome through its role in maintaining the integrity of rRNA. The existence of paralogues of RtcB with distinctive biochemical activities as seen in for example *Myxococcus xanthus* (28) suggests elaborations of RtcB functionalities will be important in some bacteria. Taken together, with the role that RtcB plays in tRNA maturation (5,6) and the unfolded protein response in higher systems (9,10), our findings suggest that RNA repair systems will support many key cellular processes ranging from maintaining the translational apparatus to control of antibiotic sensitivity and chemotactical behaviour in bacteria (this paper) to establishing neuronal networks in higher organisms (13,14).

## Supplementary Material

SUPPLEMENTARY DATA
