# Supplementary material for: Cellular and molecular phenotypes depending upon the RNA repair system RtcAB of Escherichia coli
Source: Nucleic Acids Res. 2016 Jul 8;44(20):9933–41. doi: 10.1093/nar/gkw628 (PMC5175333; doi:10.1093/nar/gkw628)
Supplement: SUPPLEMENTARY DATA [file supp_44_20_9933__index.html]

Cellular and molecular phenotypes depending upon the RNA repair system RtcAB of Escherichia coli — Cellular and molecular phenotypes depending upon the RNA repair system RtcAB of Escherichia coli — SUPPLEMENTARY DATA 

# Cellular and molecular phenotypes depending upon the RNA repair system RtcAB of *Escherichia coli*

## SUPPLEMENTARY DATA

- SUPPLEMENTARY DATA
- SUPPLEMENTARY DATA
