# Supplementary material for: Cellular and molecular phenotypes depending upon the RNA repair system RtcAB of Escherichia coli
Source: Nucleic Acids Res. 2016 Jul 8;44(20):9933–41. doi: 10.1093/nar/gkw628 (PMC5175333; doi:10.1093/nar/gkw628)

Supplementary Material

Supplementary figure legends

Supplementary figure 1: Expression regulation and growth effects of the Rtc system. (a, b) Shown are the *rtcB* and *rtcA* mRNA expression levels as quantified by RT-qPCR in *Escherichia coli* cells. **(a)** Expression levels were assessed in cells lacking *rtcA* (Δ*rtcA*) or *rtcB* (Δ*rtcB*). **(b)** Wildtype (WT) or cells lacking either *rtcR* (Δ*rtcR*) or *rtcB* (Δ*rtcB*) contained empty pBAD18cm (-), pBAD18cm expressing full-length RtcR (*rtcR),* the constitutively active $RtcR_{\Delta NTD}$ ($rtcR_{\Delta NTD}$) or full-length RtcB (*rtcB).* **(c)** Shown are growth curves (recorded as OD_{600}) of *Escherichia coli* cells in LB. Wildtype (WT) or cells lacking either *rtcA* (Δ*rtcA*) or *rtcB* (Δ*rtcB*) contained empty pBAD18cm (-), pBAD18cm expressing full-length RtcA (*rtcA)* or RtcB (*rtcB)* or the catalytic mutants $RtcA_{H308A}$ ($rtcA_{H308A}$) or $RtcB_{H337A}$ ($rtcB_{H337A}$), respectively.

Supplementary figure 2: The Rtc system helps to survive colicin D treatment. Shown are growth curves (recorded as OD_{600}) of *Escherichia coli* cells in absence (black) and presence (brown) of 250 nM elongator tRNase colicin D. Cells contained empty pBAD18cm, pBAD18cm expressing RtcA (pBAD18cm(*rtcA*)), RtcB (pBAD18cm(*rtcB*)), catalytic mutants $RtcA_{H308A}$ (pBAD18cm($rtcA_{H308A}$)) or $RtcB_{H337A}$ (pBAD18cm($rtcB_{H337A}$)).

Supplementary figure 3: Unbiased screens for Rtc inducers. (a) Abiotic compounds which increase P_{rtcBA} activity. **(b)** Shown is the effect of selected abiotic compounds on the chromosomal *gfp* expression of a constitutive GFP-overexpressing *Escherichia coli* strain. **(c)** Genetic lesions which increase P*rtcBA* activity. Shown is the fold change of P_{rtcBA} activity and *rtcBA* mRNA levels together with the beta-galactosidase activity corresponding to chromosomal *lacZ* expression of the $lacZ^+$ *Escherichia coli* strain MG1655.

Supplementary table 1: Description of Rtc inducers identified in unbiased screens.

Supplementary figure 4: The Rtc system helps to survive tetracycline treatment. Shown are growth curves (recorded as OD600) of *Escherichia coli* cells in presence of 1.5 µg/ml tetracycline added at mid exponential phase. Cells contained empty pBAD18cm, pBAD18cm expressing RtcA (pBAD18cm(*rtcA*)) or RtcB (pBAD18cm(*rtcB*)).

Supplementary figure 5: The Rtc system affects rRNA expression levels. Shown are the 16S and 23S rRNA expression levels as quantified by RT-qPCR in *Escherichia coli* cells. Wildtype (WT) or cells lacking *rtcB* (Δ*rtcB*) contained empty pBAD18cm (-) or pBAD18cm expressing full-length RtcB (*rtcB*).

Supplementary figure 1a

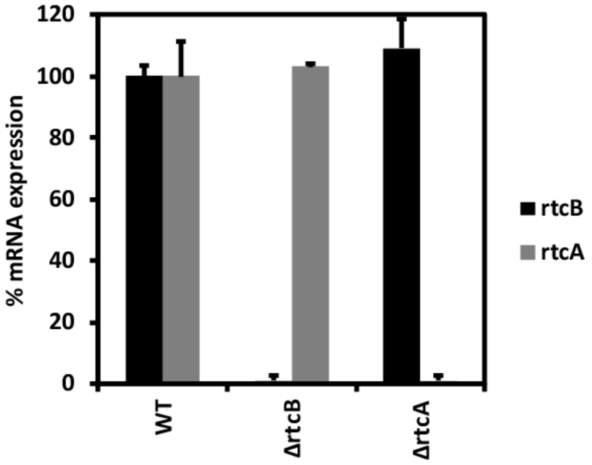

Supplementary figure 1b

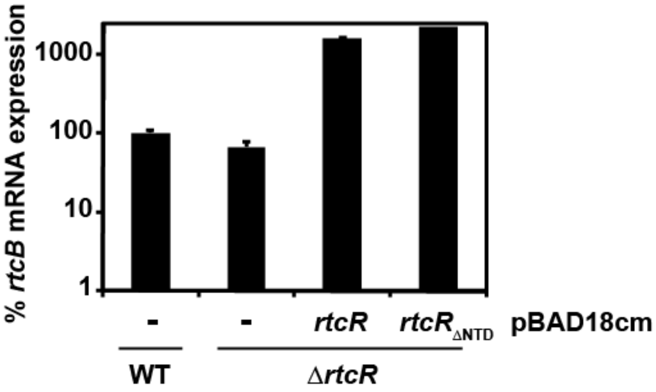

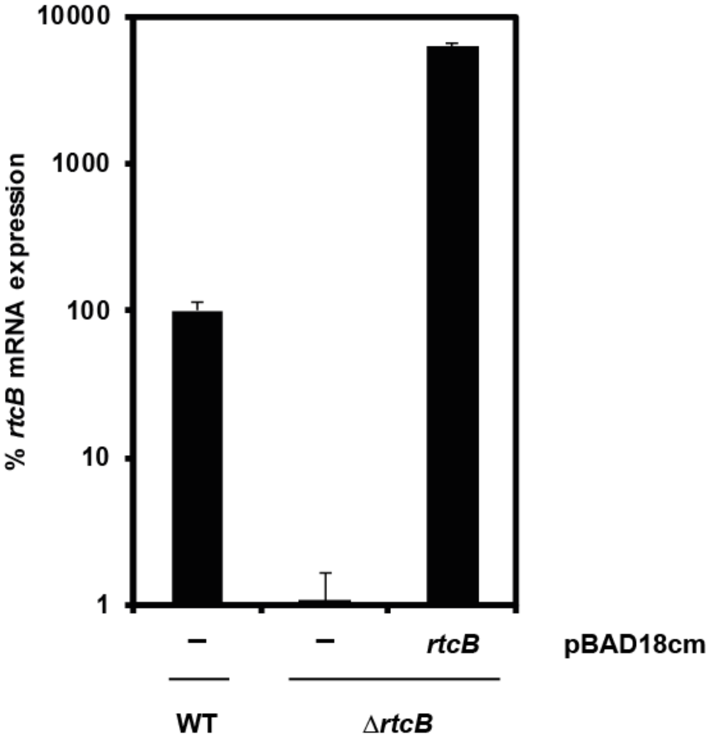

Supplementary figure 1c

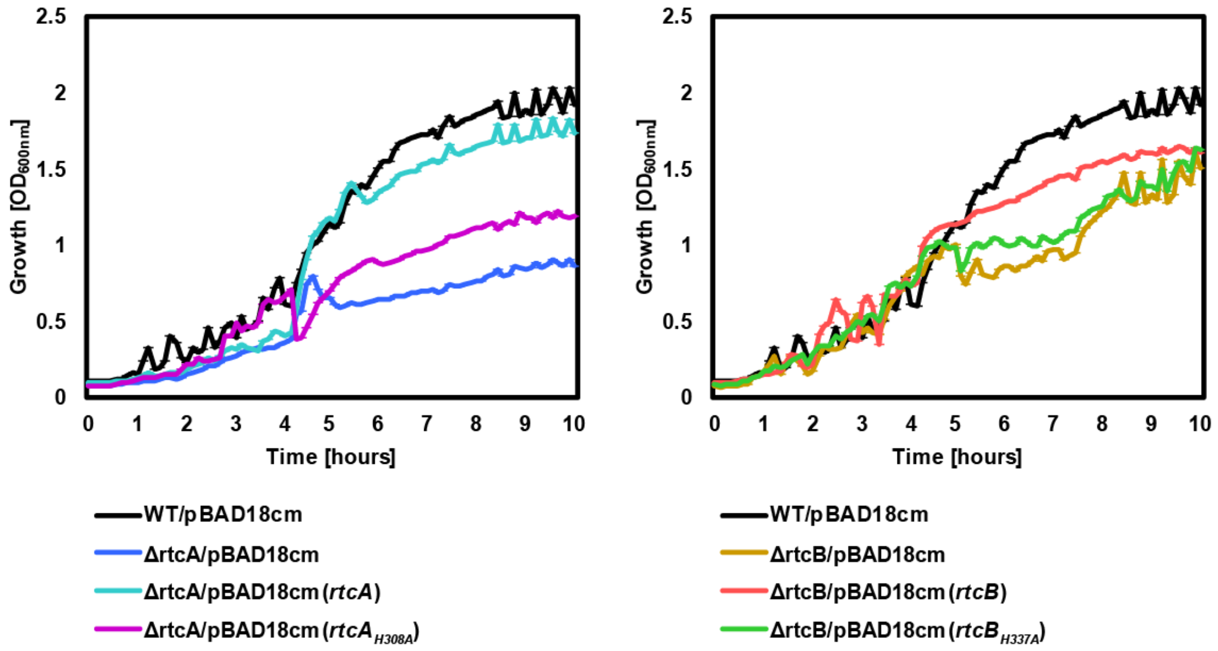

Supplementary figure 2

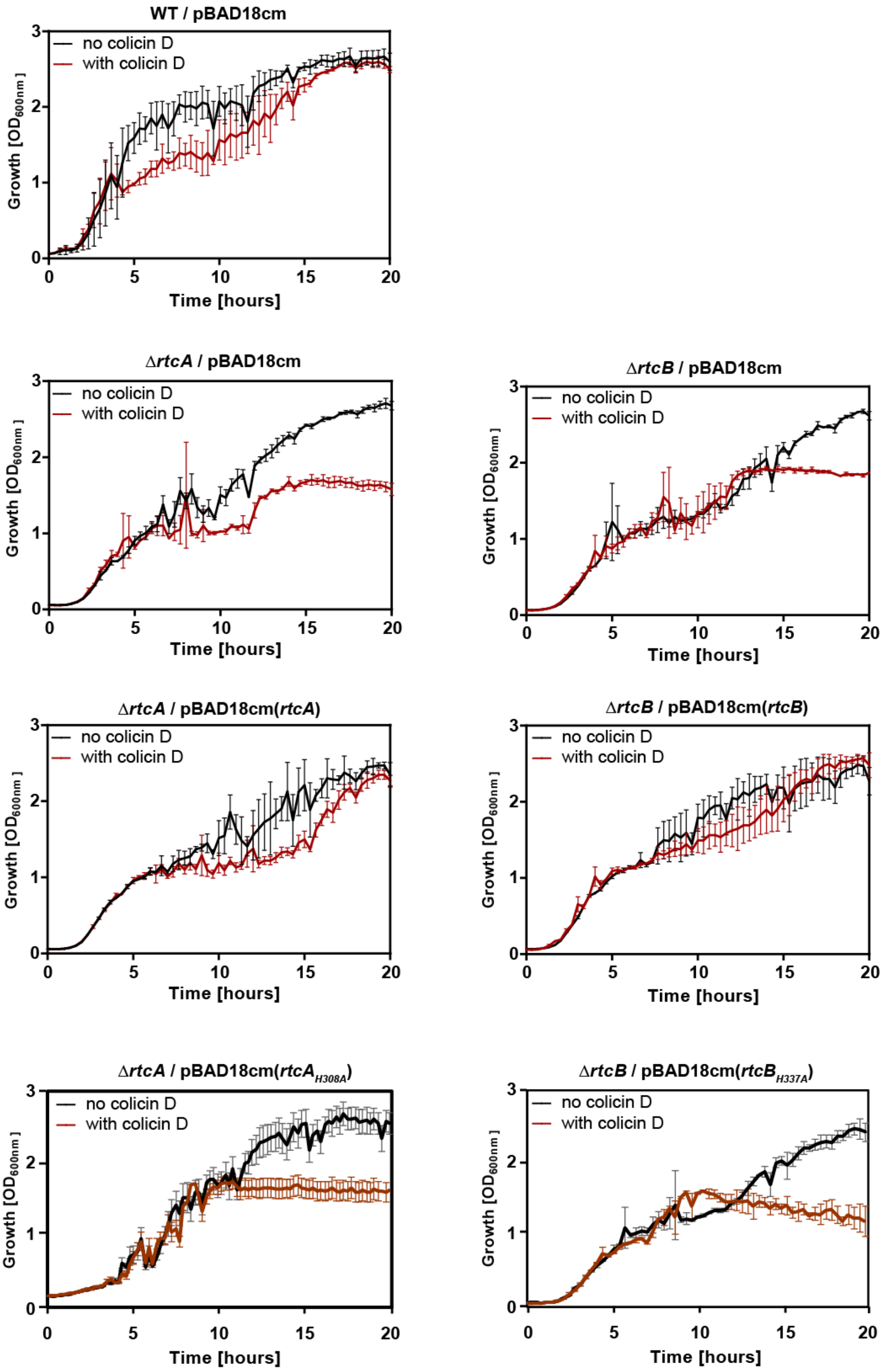

Supplementary figure 3a

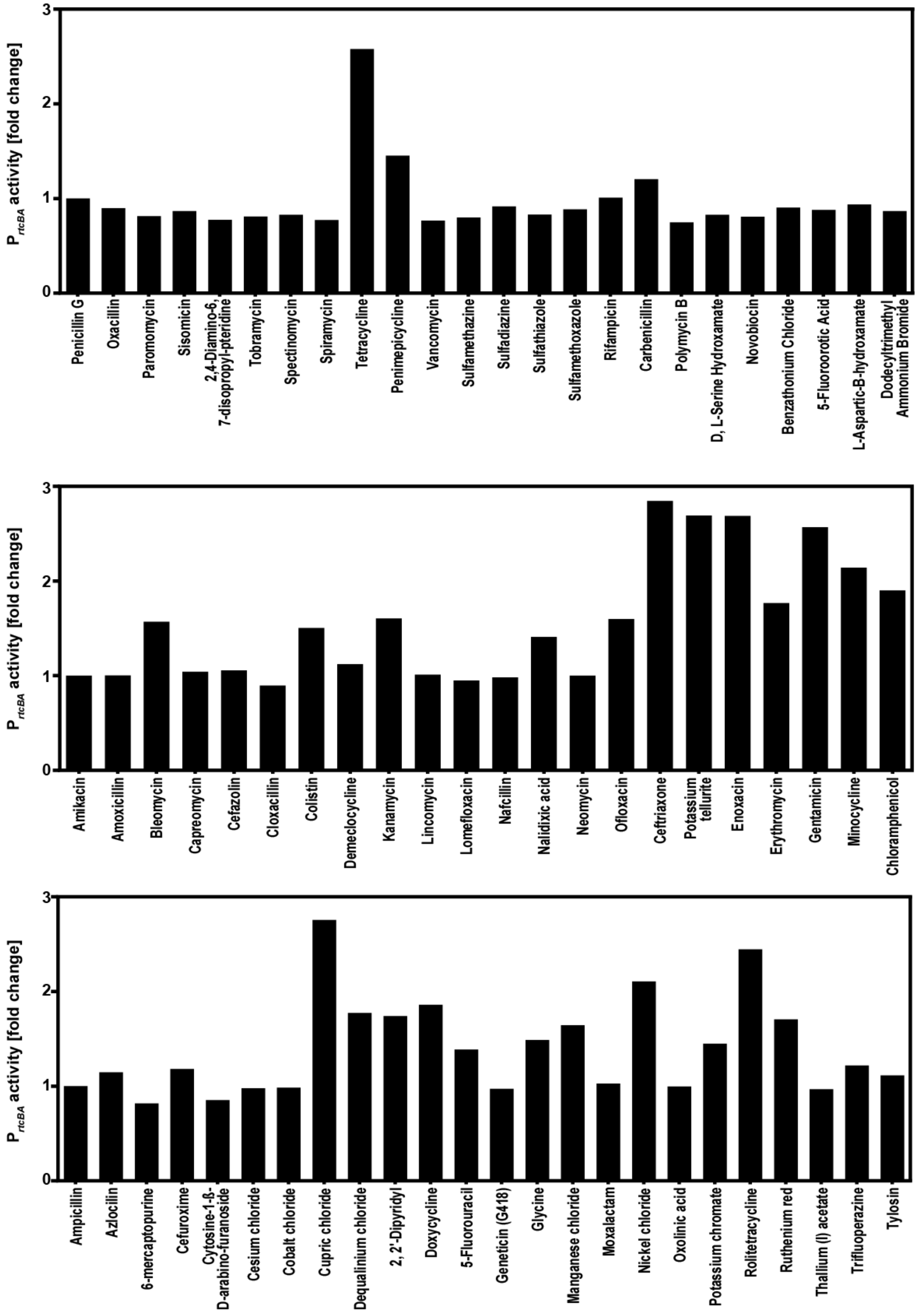

Supplementary figure 3b

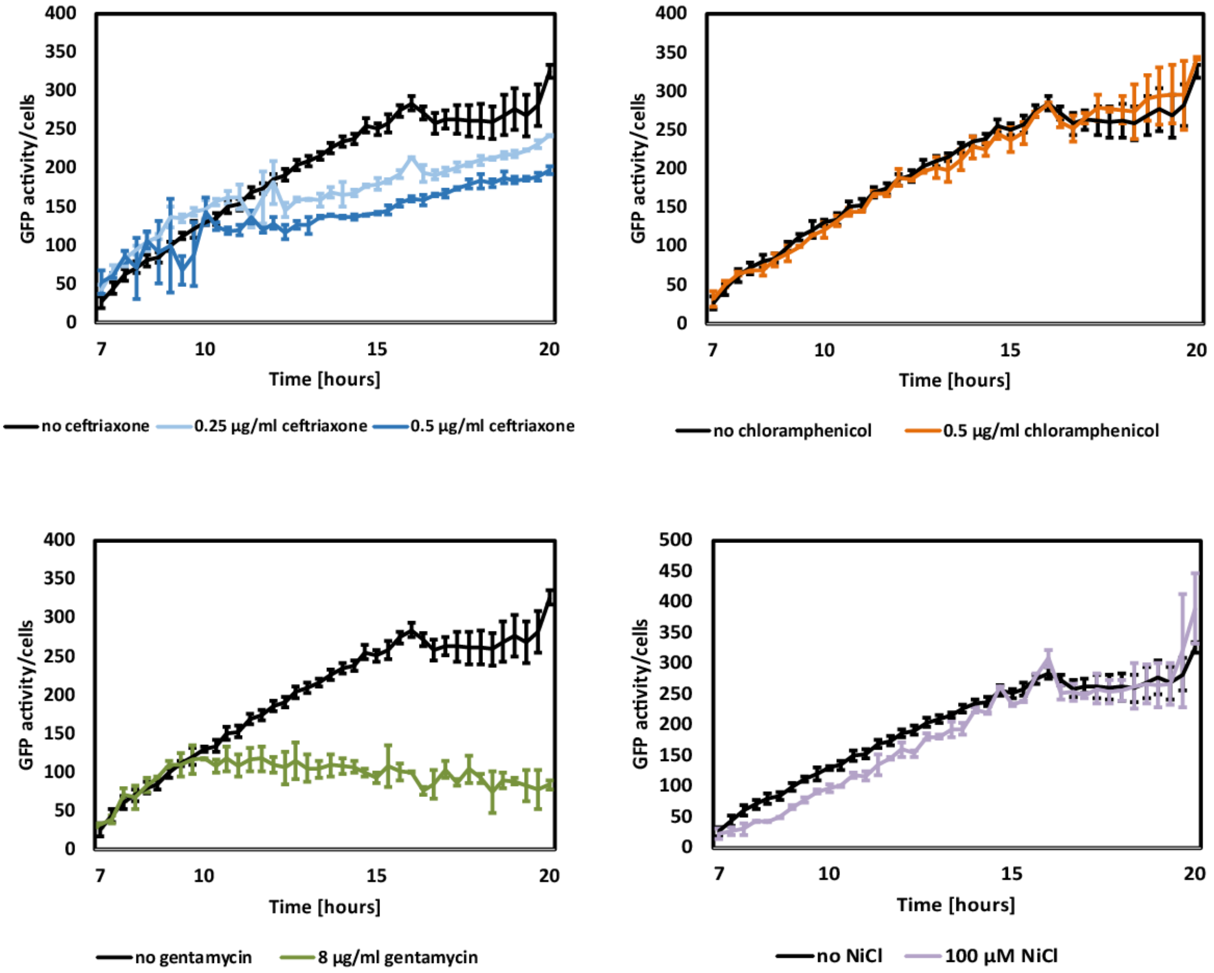

Supplementary figure 3c

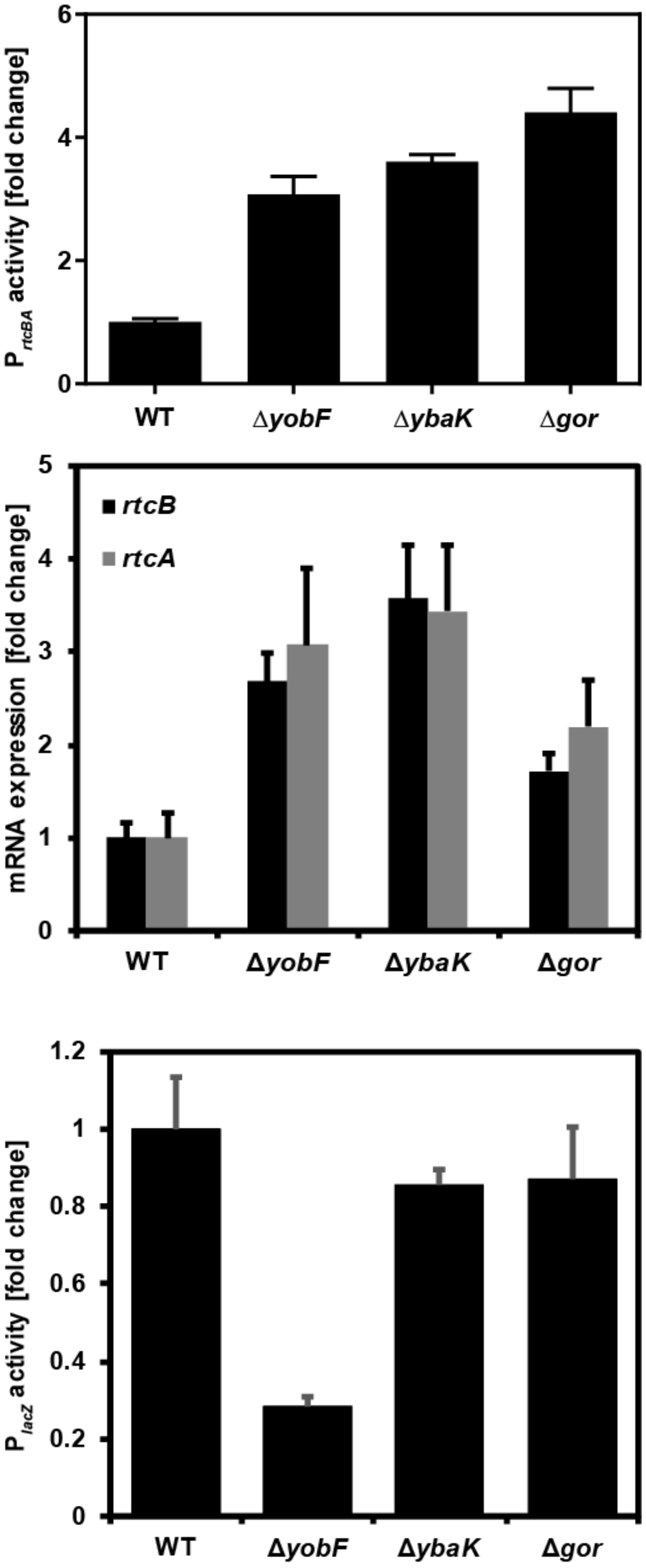

Supplementary table 1

**P*rtcBA* inducer**	**Description**
**Abiotic compounds**	
Tetracycline	antibiotic; binds to 16S rRNA and prevents amino-acyl tRNA from binding to the A-site within the ribosome
Ceftriaxone	antibiotic; inhibits cell wall biosynthesis by mimicking D-alanyl-D-alanine
Potassium tellurite	generates superoxide radicals (29)
Enoxacin	antibiotic; inhibits DNA gyrase and topoisomerase IV
Gentamicin	antibiotic; binds to A-site of 16S rRNA causing mRNA misreading and inhibition of ribosome-translocation.
Minocycline	tetracycline derivative
Chloramphenicol	antibiotic; binds to A-site of 23S rRNA preventing peptide-bond formation
Cupric chloride	redox-active metal ion causing oxidative stress (30)
Nickel chloride	nickel inhibits superoxide dismutase (31)
Rolitetracycline	tetracycline derivative
**Genetic lesions**	
yobF	stress-induced peptide; in operon with ribosome-associated CspC
ybaK	Cys-$tRNA^{Pro}$ and Cys-$tRNA^{Cys}$ deacylase; tRNA editing
gor	GSH oxidoreductase; in operon with 23S rRNA methyl-transferase

Supplementary figure 4

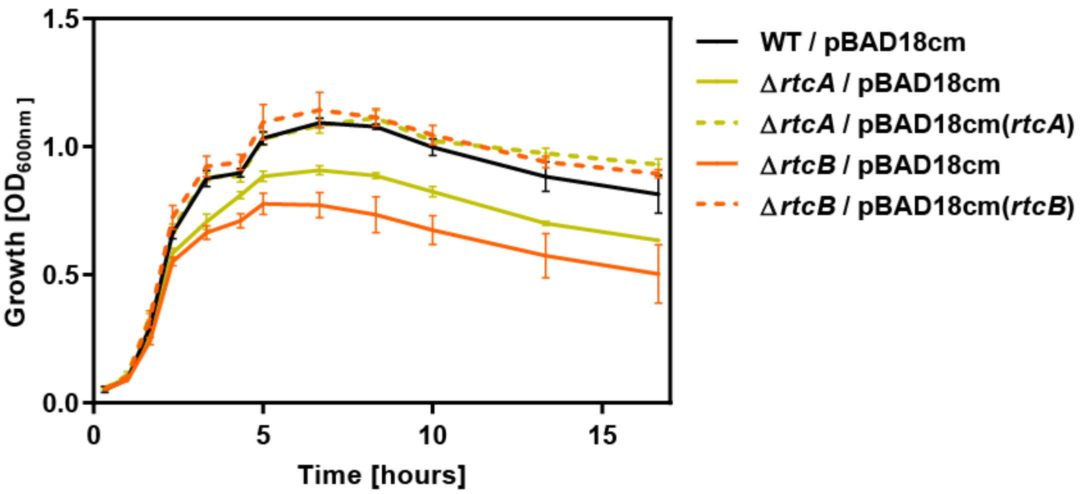

Supplementary figure 5

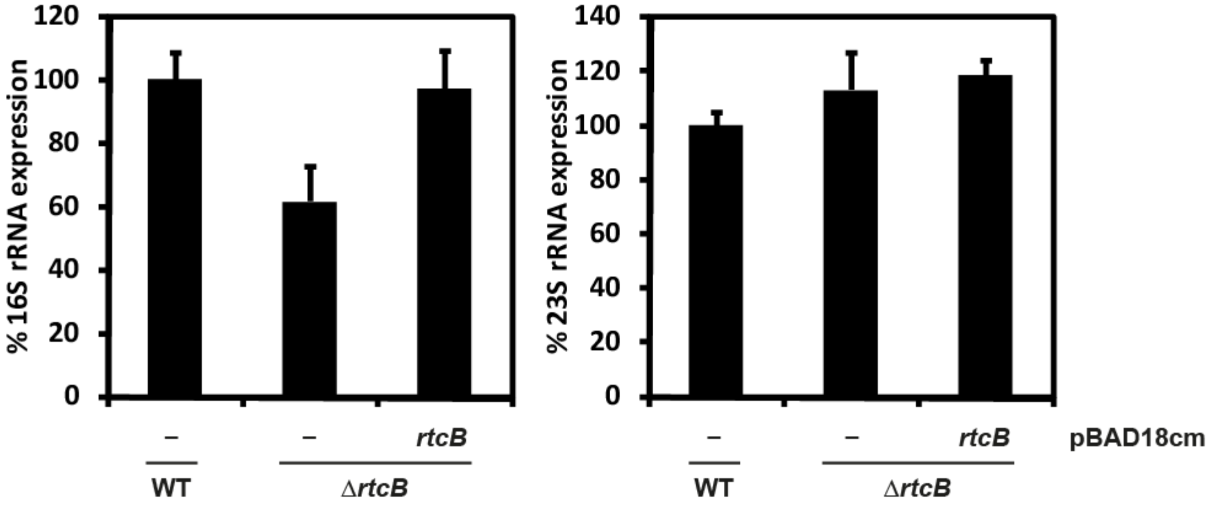

Supplement: SUPPLEMENTARY DATA [file supp_gkw628_nar-00470-z-2016-File002.pdf]
